# Toxicarioside O induces protective autophagy in a sirtuin-1-dependent manner in colorectal cancer cells

**DOI:** 10.18632/oncotarget.17189

**Published:** 2017-04-18

**Authors:** Yong-Hao Huang, Yan Sun, Feng-Ying Huang, Yue-Nan Li, Cai-Chun Wang, Wen-Li Mei, Hao-Fu Dai, Guang-Hong Tan, Canhua Huang

**Affiliations:** ^1^ Key Laboratory of Tropical Diseases and Translational Medicine of the Ministry of Education & Hainan Provincial Key Laboratory of Tropical Medicine, Hainan Medical College, Haikou 571199, China; ^2^ Institute of Tropical Bioscience and Biotechnology, Chinese Academy of Tropical Agricultural Sciences, Haikou 571199, China; ^3^ State Key Laboratory of Biotherapy and Cancer Center, West China Hospital, Sichuan University, Chengdu 610041, China

**Keywords:** colorectal cancer, toxicarioside O (TCO), autophagy, apoptosis, sirtuin-1 (SIRT1)

## Abstract

Colorectal cancer is the most common cancer. It has high morbidity and mortality worldwide, and more effective treatment strategies need to be developed. Toxicarioside O (TCO), a natural product derived from *Antiaris toxicaria*, has been shown to be a potential anticancer agent. However, the molecular mechanisms involved remain poorly understood. In this study, our results demonstrated that TCO can induce both apoptosis and autophagy in colorectal cancer cells. Moreover, TCO-induced autophagy was due to the increase of the expression and activity of the enzyme sirtuin-1 (SIRT1), and subsequent inhibition of the Akt/mTOR pathway. Inhibition of SIRT1 activity by its inhibitor, EX-527, attenuated TCO-induced autophagy. Of interest, inhibition of autophagy by chloroguine, an autophagy inhibitor, enhanced TCO-induced apoptotic cell death, suggesting that autophagy plays a protective role in TCO-induced apoptosis. Together, these findings suggest that combination of TCO and autophagy inhibitor may be a novel strategy suitable for potentiating the anticancer activity of TCO for treatment of colorectal cancer.

## INTRODUCTION

Colorectal cancer is the third most common cancer in men and the second most common cancer in women worldwide. There are almost 1.3 million new cases diagnosed annually and an estimated 694,000 deaths each year [1]. Currently, treatment of these patients is usually based on surgery associated with adjuvant chemotherapy, but this therapy is highly toxic and provides only modest results in advanced-stage patients [2, 3]. There is an urgent need for more effective therapeutic strategies.

**Figure 1:**
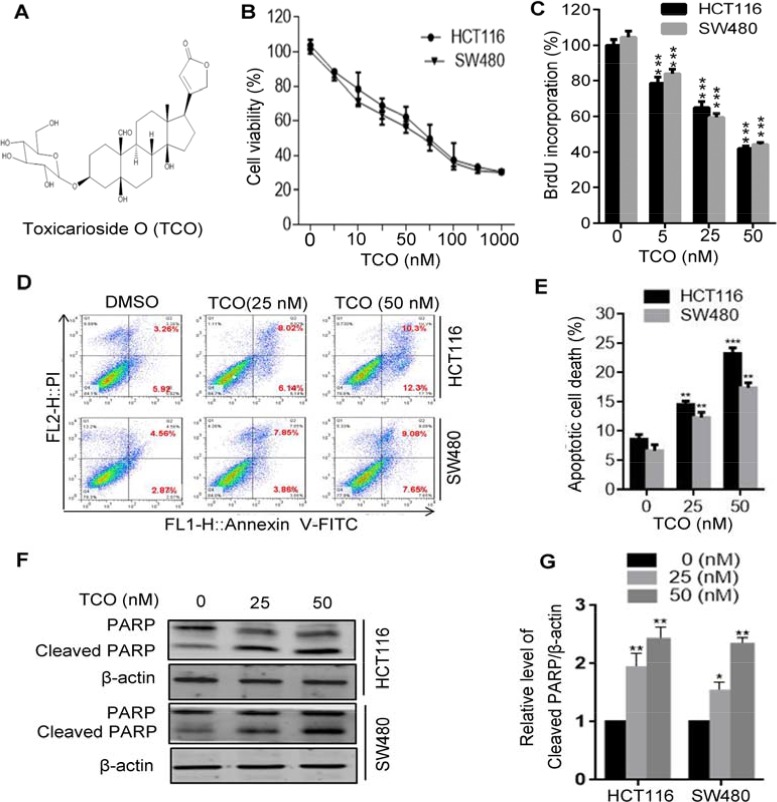
Toxicarioside O promotes apoptotic cell death in colorectal cancer cells **(A)** The structure of toxicarioside O (TCO). **(B)** Cell viability was measured using an MTT assay in HCT116 and SW480 cells treated with the indicated concentrations of TCO for 24 h. **(C)** Cells were treated with the indicated concentrations of TCO for 24 h, and the degree to which TCO inhibited cell proliferation was measured using BrdU labeling. (**D** and **E**) HCT116 and SW480 cells were treated with the indicated concentrations of TCO for 24 h. The level of apoptosis was determined using an Annexin-V-FITC/PI double staining assay. **(F)** Cells were treated as in D. The level of cleavage of PARP was determined using Western blotting. **(G)** ImageJ densitometric analysis of the Cleaved PARP/β-actin ratios from immunoblots. *P < 0.05, **P < 0.01, ***P < 0.001.

Toxicarioside O (TCO) is a natural product isolated from the seeds of *Antiaris toxicaria*. TCO is a cardenolide with a special structure (Figure 1A) that was first reported by our cooperative laboratory [4]. Traditionally, cardenolides are used clinically for the management of congestive heart failure and arrhythmia [5, 6]. Recently, increasing evidence has shown that cardenolides have anticancer activity in many cancer cell lines, acting by inhibiting sodium pumps [7–9]. Consistent with these observations, we demonstrated that TCO exhibited significant cytotoxicity against SMMC-7721 and K562 [4]. However, the underlying mechanisms by which TCO inhibits tumor growth remain poorly defined.

Autophagy is an evolutionarily conserved catabolic process in which proteins of cytosol and organelles are sequestered into a double membrane vesicle called an autophagosome, which fuses with lysosomes to form an autolysosome in which the sequestered contents are degraded or recycled [10]. Autophagy can be stimulated in response to multiple cellular stresses, such as nutrient shortage, hypoxia, and chemotherapy. Various signaling pathways have been implicated in the regulation of autophagy. One of them is the classic Akt/mTOR pathway, which negatively regulates autophagy [11]. Autophagy has been observed in a range of types of cancer cells challenged with intra- and extra-cellular stress [12, 13]. For tumor development, autophagy eliminates sources of cellular damage and recycles materials and energy to protect the cells from stress induced by chemotherapy or nutrient shortage. Hence, autophagy plays a protective role in tumor development [14, 15]. However, increasing evidence suggests that autophagy can induce tumor cell death and plays a tumor-suppressive role [16, 17]. In this way, understanding the roles of autophagy and the regulated signaling pathways involved in cancer cells is important to targeting autophagy as cancer therapy.

In this study, we demonstrated that TCO induced protective autophagy in colorectal cancer cells. TCO-induced autophagy was mediated by the upregulation of SIRT1 and subsequent inhibition of the Akt/mTOR pathway. These findings provide the groundwork for future studies on the implication of autophagy in TCO-mediated anticancer activities.

## RESULTS

### Toxicarioside O promotes apoptotic cell death in colorectal cancer cells

To investigate the effect of TCO on colorectal cancer growth, human colorectal cancer cell lines HCT116 and SW480 cells were treated with various concentrations of TCO for 24 h, followed by MTT assay. As shown in Figure 1B, the cell viability decreased in a dose-dependent manner in both cell lines. In addition, a BrdU cell proliferation assay showed significantly lower percentage of BrdU-positive cells in TCO-treated cells but not in the controls (Figure 1C). These results demonstrated that TCO markedly suppressed colorectal cancer cell proliferation. To determine whether TCO promotes cell death by inducing apoptosis in colorectal cancer cells, cells were analyzed by flow cytometry following Annexin V-FITC and propidium iodide (PI) staining. As shown in Figure 1D and 1E, TCO treatment for 24 h significantly increased the percentage of apoptotic cells over control cultures. Consistent with this observation, the level of cleaved PARP was higher in TCO-treated cells than in controls (Figure 1F and 1G). Collectively, these data demonstrated that TCO promotes apoptotic cell death in colorectal cancer cells.

### Toxicarioside O induces autophagy in colorectal cancer cells

**Figure 2:**
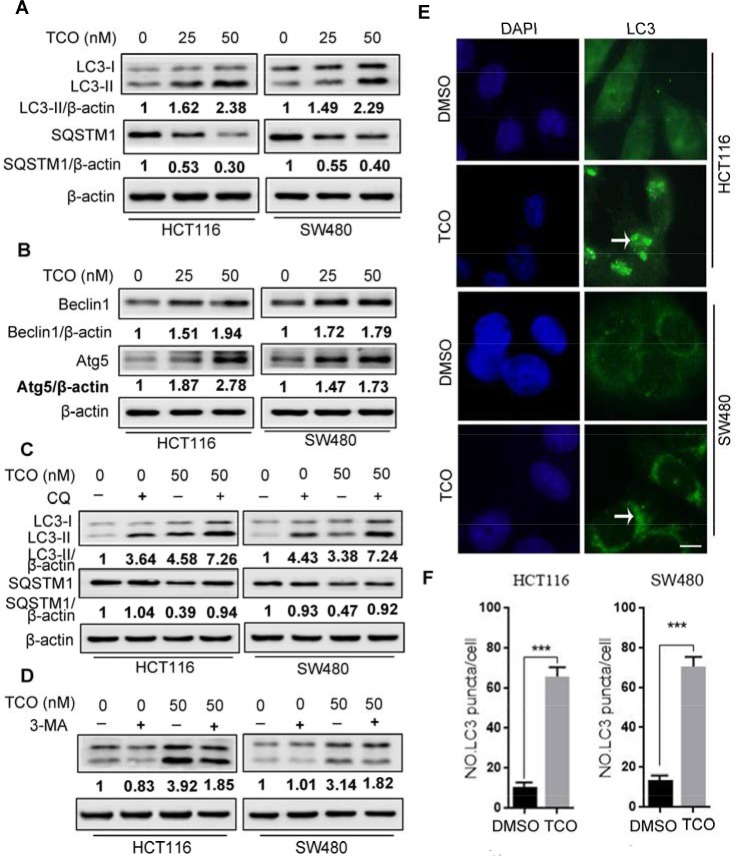
Toxicarioside O induces autophagy in colorectal cancer cells **(A)** Immunoblot analysis of LC3 and SQSTM1 in cells treated with the indicated concentrations of TCO for 24 h. Protein ratios were calculated following ImageJ densitometric analysis. **(B)** Immunoblot analysis of Atg5 and Beclin 1 in cells treated as in A. Protein ratios were calculated following ImageJ densitometric analysis. **(C)** Immunoblot analysis of LC3 and SQSTM1 in cells treated with the indicated concentrations of TCO for 24 h in the absence or presence of chloroguine (CQ). Protein ratios were calculated following ImageJ densitometric analysis. **(D)** Immunoblot analysis of LC3 in cells treated with the indicated concentrations of TCO for 24 h in the absence or presence of 3-methyladenine (3-MA). (**E** and **F**) HCT116 and SW480 cells were treated with DMSO or 50 nM of TCO for 24 h, the formation of endogenous LC3 puncta was visualized under a fluorescent microscope. Compared with the DMSO-treated cells, TCO-treated cells were shrinked and the LC-3 puncta seem to clump together (arrows). Scale bar: 10 μm. ***P < 0.001.

Because autophagy has been considered as a target for anticancer therapy [12, 18], we next addressed whether TCO induces autophagy in colorectal cancer cells. We first detected the conversion of LC3-I to lipidated LC3-II and the distribution of endogenous LC3 puncta, two classical markers of autophagy. As shown in Figure 2A, 2E and 2F, TCO treatment markedly increased LC3-II conversion and LC3 puncta in colorectal cancer cells. We also examined the expression of Beclin 1 and Atg5, two autophagy-related proteins. As shown in Figure 2B, TCO elevated the expression of both Beclin1 and Atg5 in a dose-dependent manner. Collectively, these results demonstrated that TCO induces autophagy in colorectal cancer cells. To determine whether TCO could alter the detection of autophagy, we tested the SQSTM1 protein concentration in cells, a well-known autophagy substrate that is degraded through the autophagy pathway. As shown in Figure 2A, TCO treatment resulted in degradation of SQSTM1 in a dose-dependent manner. Combinatorial treatment with chloroquine (CQ, a lysosomal inhibitor) resulted in the accumulation of SQSTM1 and LC3-II conversion (Figure 2C). In addition, combinatorial treatment with 3-methyladenine (3-MA, an autophagy inhibitor) markedly decreased the LC3-II conversion in TCO-treated cells (Figure 2D). These results showed that LC3-II conversion was higher in the TCO-treated cells than in the controls in both the presence and absence of CQ (Figure 2A and 2C). Taken together, these results suggested that TCO promoted autophagic flux in colorectal cancer cells.

### Toxicarioside O induces autophagy through inhibition of the Akt/mTOR pathway

**Figure 3:**
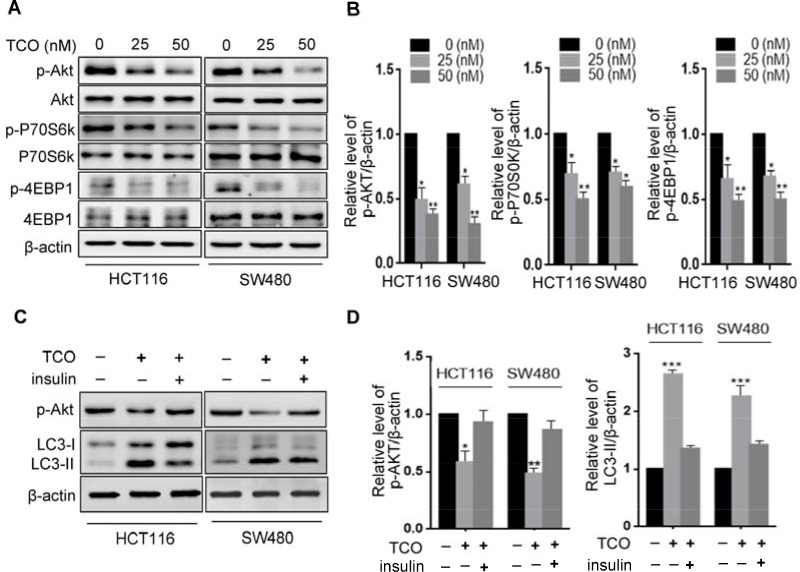
Toxicarioside O induces autophagy through inhibition of the AKT1/mTOR pathway **(A)** Immunoblot analysis of phosphorylation of Akt (Ser473), P70S6K(Ser424 and Thr421), and 4EBP1 (Ser65 and Thr70) in cells treated with the indicated concentrations of TCO for 24 h. Total Akt, p70S6K, and 4EBP1 expression served as internal controls. **(B)** ImageJ densitometric analysis of the p-AKT/β-actin, the p-P70S6K/β-actin, and the 4EBP1/β-actin ratios from immunoblots. **(C)** Immunoblot analysis of LC3 and p-Akt in cells treated with 50 nM of TCO for 24 h in the presence and absence of insulin. **(D)** ImageJ densitometric analysis of the p-AKT/β-actin and the LC3-II/β-actin ratios from immunoblots. *P < 0.05, **P < 0.01, ***P < 0.001.

The Akt/mTOR pathway is considered a major negative regulator of autophagy [11, 19]. We determined whether the Akt/mTOR pathway was inhibited in TCO–treated colorectal cancer cells. First, we examined the phosphorylation status of Akt by Western blot. As shown in Figure 3A and 3B, TCO significantly decreased the phosphorylation levels of Akt (Ser473). Then, we determined the phosphorylation status of p70S6K (Ser424 and Thr421), and 4E-BP1 (Ser65 and Thr70), two best characterized targets of mTOR1 complex. As shown in Figure 3A and 3B, TCO also markedly decreased the phosphorylation levels of p70S6K and 4E-BP1. These results indicated that TCO inhibited the Akt/mTOR pathway in colorectal cancer cells. To further determine whether inhibition of the Akt/mTOR pathway is involved in TCO–induced autophagy, we activated the Akt/mTOR pathway by combinatorial treatment with insulin (Akt activator) in colorectal cancer cells. As shown in Figure 3C and 3D, Akt activation by insulin produced markedly lower levels of LC3-II conversion in the TCO-treated cells than in the controls, suggesting that TCO induced autophagy in colorectal cancer cells by inhibition of the Akt/mTOR pathway.

### Toxicarioside O induces autophagy through regulation of SIRT1 in colorectal cancer cells

**Figure 4:**
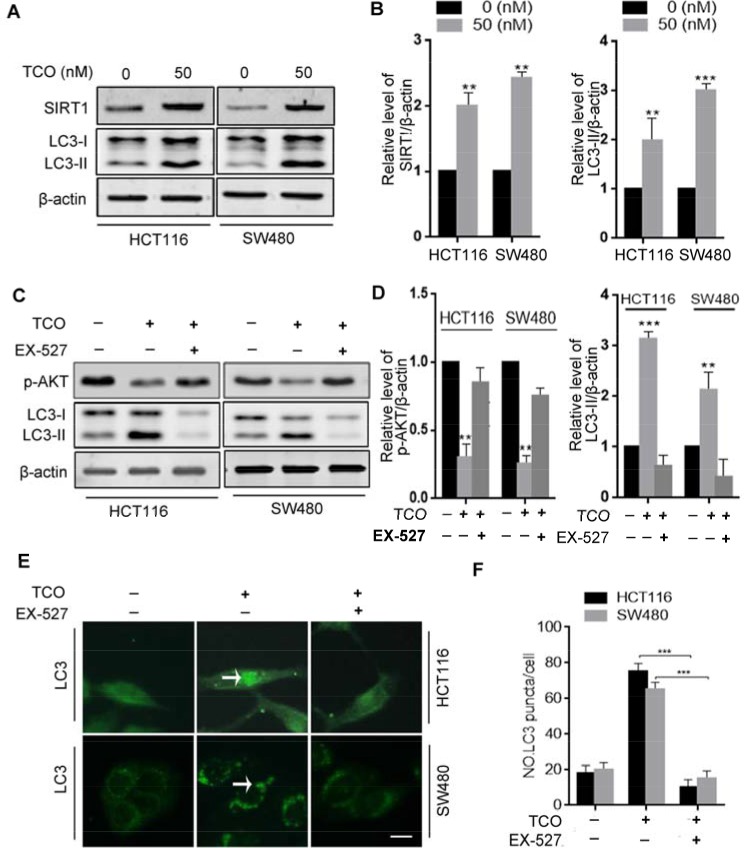
Toxicarioside O induces autophagy through up-regulation of SIRT1 in colorectal cancer cells **(A)** Immunoblot analysis of sirtuin-1 (SIRT1) and LC3 in cells treated with the indicated concentrations of TCO for 24 h. **(B)** ImageJ densitometric analysis of the SIRT1/β-actin and the LC3-II/β-actin ratios from immunoblots. **(C)** Immunoblot analysis of p-AKT (Ser473) and LC3-II in cells treated with 50 nM of TCO for 24 h in the presence and absence of EX-527. **(D)** ImageJ densitometric analysis of the p-AKT/β-actin and the LC3-II/β-actin ratios from immunoblots. (**E** and **F**) Cells were treated as in B. The formation of endogenous LC3 puncta (arrows) was visualized under a fluorescent microscope. Scale bar: 10 μm. **P < 0.01, ***P < 0.001.

It has previously been reported that SIRT1 induces autophagy through inhibition of mTOR signaling [20, 21]. For this reason, we next addressed whether SIRT1 is involved in TCO-induced autophagy in colorectal cancer cells. First, we determinedthe expression of SIRT1 in TCO-treated cells. As shown in Figure 4A and 4B, TCO treatment promoted SIRT1 expression in colorectal cancer cells and increased LC3-II conversion. SIRT1 is an NAD-dependent deacetylase and it regulates autophagy by its enzymatic activity [22, 23]. As expected, inhibition of SIRT1 activity by its inhibitor, EX-527, attenuated TCO-induced autophagy, as evidenced by decreased LC3-II conversion and LC3 puncta in colorectal cancer cells (Figure 4C-4F). Moreover, inhibition of SIRT1 activity by EX-527 reverted the phosphorylation levels of Akt (Ser473) and markedly suppressed LC3-II conversion in the TCO-treated cells (Figure 4C and 4D), suggesting that TCO blocked Akt/mTOR pathway through SIRT1. Collectively, these results indicated that TCO-induced autophagy in colorectal cancer cells was mediated by the upregulation of SIRT1.

### Inhibition of autophagy enhances the TCO-induced anti-proliferative effect

**Figure 5:**
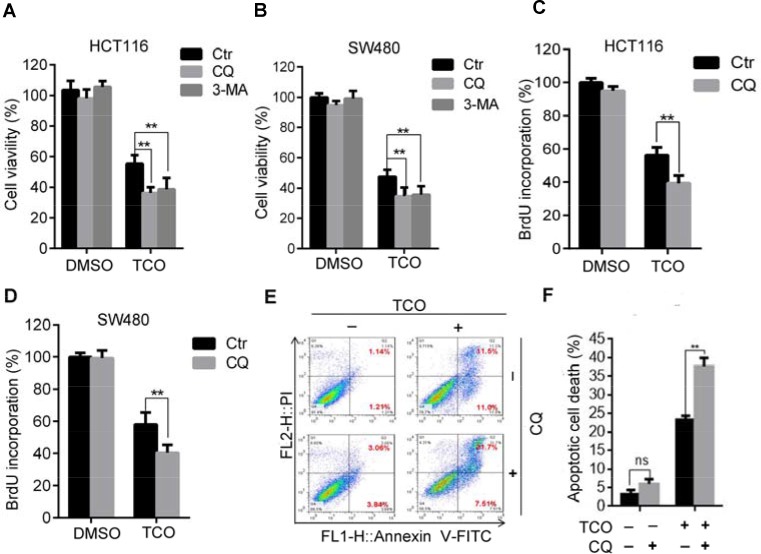
Inhibition of autophagy enhances the TCO-induced anti-proliferative effect (**A** and **B**) Cell viability was measured by MTT assay in cells treated with DMSO or 50 nM of TCO for 24 h in the presence and absence of CQ or 3-MA. (**C** and **D**) Cell proliferation was measured using BrdU labeling in cells treated with DMSO or 50 nM of TCO for 24 h in the presence and absence of CQ. (**E** and **F**) HCT116 Cells were treated as in B. Apoptosis was determined using an Annexin-V-FITC/PI double staining assay. **P < 0.01.

Because TCO promotes apoptotic cell death as it induces autophagy in colorectal cancer cells, and because accumulating evidence suggested autophagy has dual roles in cell survival and cell death [15, 24], we next addressed the role of autophagy in TCO-induced apoptotic cell death. As shown in Figure 5A and 5B, combinatorial treatment with autophagic inhibitor, CQ or 3-MA resulted in significantly lower colorectal cell viability than TCO treatment alone. Consistently, similar results were observed by BrdU incorporation assay in combinatorial treatment of CQ or in colorectal cancer cells (Figure 5C and 5D). Furthermore, Annexin-V/PI staining also demonstrated that combinatorial treatment of CQ enhanced TCO-induced apoptotic cell death (Figure 5E and 5F). These findings suggested that TCO-induced autophagy plays a protective role against TCO-induced apoptosis in colorectal cancer.

## DISCUSSION

*Antiaris toxicaria* is well known as the “arrow poison wood” because its latex contains a complex, toxic mixture of cardenolide glycosides used as poisons for arrows and darts in many countries [25, 26]. In addition to the traditional effect on congestive heart failure and arrhythmia, cardenolide glycosides have been shown to have anticancer activities in various cancer cell lines. In recent years, our research group has isolated several new cardenolides from the latex, seeds, and stem of *Antiaris toxicaria* and these have been proved to possess significant cytotoxicity against SGC-7901, SMMC-7721, K562 and HeLa cell lines [27–30]. Toxicarioside O (TCO), a cardenolide isolated from the seeds of *Antiaris toxicaria*, has been shown to have potential anticancer activities [4]. However, the molecular mechanisms underlying TCO-mediated suppression of tumor still remain poorly understood. In this study, we demonstrated that TCO inhibits cell growth and induces apoptosis in human colorectal cancer cell lines HCT116 and SW480. In addition, our results also showed that TCO induces autophagy in these colorectal cancer cells, suggesting that both apoptosis and autophagy are involved in TCO-mediated suppression of cancer.

Accumulating evidence has indicated that many anticancer agents can induce autophagy in cancer cells [31, 32], but the role that autophagy plays in cancer therapy is still unclear. Generally, autophagy plays a pro-survival role in cancer cells by removing damaged organelles and recycling nutrients upon anticancer treatment [14, 15]. However, growing amounts of evidence have demonstrated that certain anticancer agents induce autophagic cell death and so enhance chemotherapeutic efficacy in cancer therapy [33–35]. In this study, our findings indicated that TCO triggered autophagy in human colorectal cancer cells, and the disruption of autophagy using autophagic inhibitor CQ or 3-MA increased TCO-induced apoptosis. Thus, our results showed in the current study indicate that TCO-induced autophagy plays a pro-survival or protective role in colorectal cancer cells, and the combinatory use of autophagic inhibitor such as CQ may possibly increase the treatment effectiveness induced by TCO.

Multiple signaling pathways have been shown to regulate autophagy. The Akt/mTOR pathway is a well-known major negative regulator of autophagy [11, 19]. In this study, we investigated whether Akt/mTOR pathway is involved in the autophagic process induced by TCO. Our results showed that the Akt/mTOR pathway was markedly inhibited by TCO. Notably, our results demonstrated that TCO significantly decreased the phosphorylation levels of Akt, p70S6K, and 4E-BP1 in colorectal cancer cells. Reactivation of the Akt/mTOR pathway by insulin substantially abolished TCO-induced autophagy. These data strongly indicated that the functional importance of the Akt/mTOR pathway in TCO-induced autophagy in colorectal cancer cells.

Sirtuin-1 (SIRT1), an NAD-dependent deacetylase, plays a regulatory role in autophagy. Sirt1 can deacetylate Atg5, Atg7, and LC3 and directly participates in the regulation of autophagy [22, 23]. In addition, Sirt1 controls autophagy by deacetylation of the transcription factors that regulate the expression of autophagy-associated genes [36, 37]. SIRT1 induces autophagy through negative regulation of mTOR signaling [21, 38]. In this study, our results demonstrated that TCO treatment increased SIRT1 expression in colorectal cancer cells, and inhibition of SIRT1 activity by EX-527 attenuated TCO-induced autophagy, suggesting that SIRT1 plays a critical role in TCO-induced autophagy in colorectal cancer cells.

In summary, we demonstrated that TCO induced autophagy via the upregulation of SIRT1 in colorectal cancer cells. The disruption of autophagy by CQ increased the rate of TCO-induced cell death, suggesting that TCO-induced autophagy plays a protective role in colorectal cells. These findings suggest that combinational treatment of autophagy inhibitors may be a promising strategy for TCO-mediated anticancer therapy.

## MATERIALS AND METHODS

### Cell culture, agents, and antibodies

Human colorectal cancer cell lines HCT116 and SW480 cells purchased from the American Type Culture Collection were cultured in DMEM, supplemented with 10% fetal bovine serum (Biowest), 100 U/ml penicillin (Sigma), 100 μg/ml streptomycin (Sigma) at 37°C in an atmosphere containing 5% CO_2_. A BrdU Cell Proliferation Assay Kit was purchased from Abcam. An Annexin V-FITC/PI Apoptosis Detection Kit was purchased from KeyGEN Biotech. Enhanced chemiluminescence solution was from Merck Millipore. Toxicarioside O (TCO) was isolated and purified from the seeds of *Antiaris toxicaria* in our laboratory [4]. The purity of TCO was proved to be ≧95% by a chromatographic analysis. TCO was dissolved in DMSO and stored at −20°C for experiment use in this study. EX-527 was obtained from MedChemExpress. CQ and 3-MA were obtained from Sigma-Aldrich. The following antibodies were used in this study: phosphorylated and total forms of Akt, Phospho-p70S6 kinase, p70S6, phospho-4E-BP1, 4E-BP1, and Atg5 were purchased from Cell Signaling Technology; SQSTM1 and Beclin1 was from Santa Cruz Biotechnology; LC3 was from Sigma-Aldrich; PARP and SIRT1 was purchased from Abcam.

### Cell viability assay

Cells were cultured in 96-well plates and exposed to the tested compounds for 24 h. The cell viabilities were determined by 3-(4,5-dimethylthiazol-2-yl)-2,5-diphenyltetrazolium bromide (MTT) assay. Briefly, 10 μl of MTT was added to each well and incubated for 4 h. The medium was removed, and 150 μl of DMSO was added to each well to dissolve the crystal formazan dye. The absorbance was measured at 570 nm on an enzyme-linked immunosorbent assay reader.

### BrdU assay

BrdU signaling was assayed using a BrdU Cell Proliferation Assay Kit (Abcam, ab126556). Briefly, cells were cultured in 96-well plates and exposed to the tested compounds for 24 h. Subsequently, 10 μM BrdU was added to each well, and samples were incubated for 12 h at 37°C. BrdU signaling was determined by measuring the absorbance at 450 nm.

### Flow cytometry

Cells were cultured and exposed to the tested compounds for 24 h, harvested by trypsinization, and washed with PBS, then resuspended and incubated in PI/Annexin-V solution (KeyGEN Biotech) for apoptosis analysis. At least 10,000 live cells were analyzed on a FACSCalibur flow cytometer. Data were analyzed using FlowJo7.6.1 software.

### Western blotting

Cells were lysed with RIPA buffer (50 mM Tris, 1.0 mM EDTA, 150 mM NaCl, 0.1% SDS, 1% Triton X-100, 1% sodium deoxycholate, 1 mM PMSF). The concentrations of protein were quantified using a BCA protein assay kit (Thermo, 23227). Proteins were size-fractionated by 12% SDS-PAGE and then transferred to PVDF membranes. After blocking, the membranes were incubated with primary antibodies at 4°C overnight and then incubated with secondary antibodies at room temperature for 1 h. The immunoreactivities were visualized by enhanced chemiluminescence reagents (Millipore, WBKLS0100).

### Immunofluorescence

Cells were fixed with 4% paraformaldehyde for 30 min, washed three times with PBS, and then incubated with 0.1% Triton X-100 for permeabilization. Cells were stained with anti-LC3B polyclonal antibody (Sigma, L7543) overnight at 4°C and then incubated with Alexa Fluor 488–conjugated goat anti-rabbit IgG (Abcam, ab150077) at room temperature for 1 h. Nuclei were stained with DAPI. Images were captured using a fluorescent microscope (OLYMPUS, BX53).

### Statistical analysis

Data analysis was performed using GraphPad Prism 6.0. Statistical differences were determined using a two-sample equal variance Student t test. Statistical significance was defined as *P < 0.05. All data shown are reported as mean ± SD.
